# The changing epidemiology of dengue in Delhi, India

**DOI:** 10.1186/1743-422X-3-92

**Published:** 2006-11-05

**Authors:** Ekta Gupta, Lalit Dar, Geetanjali Kapoor, Shobha Broor

**Affiliations:** 1 Department of Microbiology, All India Institute of Medical Sciences. New Delhi-110029, India

## Abstract

**Background:**

A major DHF outbreak occurred in Delhi in 1996. Following this another outbreak was reported in the year 2003. In the years 2004 and 2005, though no outbreak was reported, a definitely higher number of samples were received in the virology laboratory of A.I.I.M.S. from suspected cases of dengue infection. This study was designed to compare the serological and virological profiles of confirmed dengue cases in the years 2003, 2004 and 2005.

**Results:**

Out of 1820 serum samples received from suspected cases in all three years, 811 (44.56%) were confirmed as dengue infection serologically. Out of these confirmed dengue cases maximum cases, in all three years, were seen in the age group 21–30 years. There was an increase in the number of samples received in the post monsoon period (September to November) with a peak in the second and third week of October. More samples were received from DHF cases in the year 2005 than 2004 and 2003. All four dengue serotypes were seen co-circulating in the year 2003, followed by complete predominance of dengue serotype 3 in 2005.

**Conclusion:**

Epidemiology of dengue is changing rapidly in Delhi. Dengue infections are seen every year thus making it an endemic disease. After co-circulation of all serotypes in 2003, now dengue serotype 3 is emerging as the predominant serotype.

## Background

The global epidemiology of dengue fever/dengue hemorrhagic fever (DF/DHF) is changing fast [1]. The Indian encounter with this disease is interesting and intriguing. Dengue infection has been known to be endemic in India for over two centuries as a benign and self limited disease. In recent years, the disease has changed its course manifesting in the severe form as DHF and with increasing frequency of outbreaks. Delhi, a city in North India, has experienced seven outbreaks of dengue virus infection since 1967 with the last reported in 2003 [2-4]. The 1996 epidemic in India was mainly due to the virus dengue -2[2]. While in 2003 all four serotypes of dengue viruses were found in co-circulation [5]. In the following years 2004 and 2005, though, outbreaks did not occur but higher number of cases of suspected dengue infection were reported to our hospital in the similar months as that in 1996 and 2003. In this study we have compared the serological and virological profiles of the confirmed dengue cases reported to All India Institute of Medical Sciences (AIIMS) in these three years i.e. 2003, 2004, and 2005.

## Results

**Figure 1:**
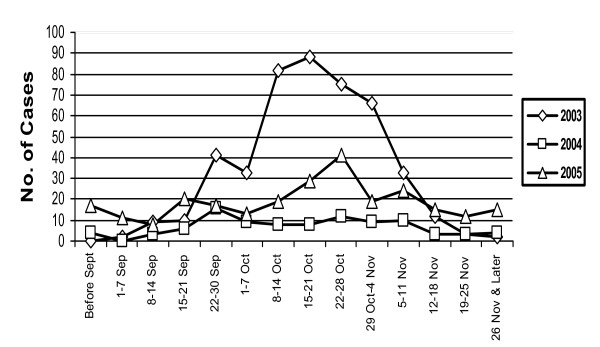
Weekly distribution of IgM positive dengue cases of all three years.

**Figure 2:**
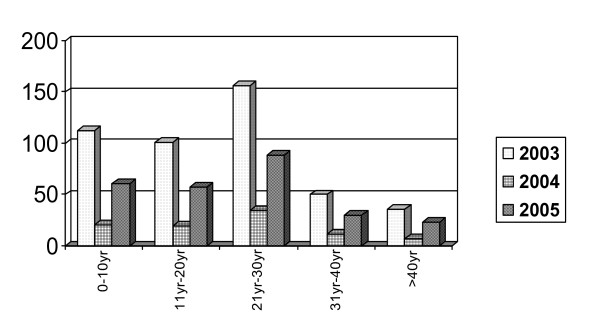
Age wise distribution of IgM positive dengue cases of all three years.

During the study period (2003–2005), a total of 1820 serum samples were tested for dengue IgM antibodies, year wise distribution of the samples being 874 in 2003, 340 in 2004 & 606 in the year 2005. Of these 811 (44.56%) were positive for dengue specific IgM antibodies. Year-wise distribution of dengue IgM positive cases over 3 year period is shown in Table 1. Maximum numbers of samples were received in the year 2003. Out of 1820 samples received 868 were from indoor patients with overall mortality of 4.14% in these indoor patients only. Year wise mortality rates in indoor patients were 4.17% in 2003, 4.9% in 2004 and 3.6%, in 2005 (not showing any significant change). Overall males predominated over females (M: F ratio) Year wise distribution of cases show male were more frequently affected as compared to females. Month-wise and week wise distribution of positive cases in all the three years (fig 1) have shown a peak in the 2^nd ^and 3^rd ^week of October. Age-wise distribution of IgM positive cases in all three years (fig. 2) clearly indicates that older age groups (>10 years) were more commonly affected than the age group ≤ 10 years (p < 0.001). Age group most commonly affected in all three years being 21–30 yrs. Clinically, percentages of dengue hemorrhagic fever (DHF) in confirmed dengue cases has shown a linear trend and were significantly more in 2005 than in the years 2004 and 2003 (p < 0.001).(Table 1)

**Table 1:** Demographic Profile of Serologically confirmed cases

	2003	2004	2005
IgM positive Cases	456	95	260
DHF Cases	47(10.3%)*	10(10.5%)*	62(23.8%)*
Male: Female ratio	2.3:1	1.7:1	1.9:1
≤ 10 Years	113	21	61
> 10 Years	343*	74*	199*

* p-value significant < 0.001

Eighty five serum samples were processed for virus isolation, followed by IFA for serotype identification (Table 2). Samples received in the year 2004 and 2005 were also subjected to multiplex RT-PCR. In the year 2003, only culture confirmed cases were further subjected to RT-PCR for re-confirmation of the serotype. All four serotypes of dengue -1, 2, 3, 4 were co-circulating in 2003[4]. In 2004 only four samples were received for virus isolation, two were positive for dengue virus by culture and RT-PCR and both were identified as dengue-1. In 2005, predominant serotype obtained was dengue-3 and apart from dengue-3 only dengue-1 was the other serotype seen. In 2005, five cases were culture positive and all were identified as dengue-3 by Indirect immunofluorescence assay (IFA) and all five patients had DHF. While by RT-PCR dengue virus RNA was detected in 17 samples of which 14 were dengue-3, 1 DEN1 and 2 samples had dual infection with dengue-1 and dengue-3 serotypes.

**Table 2:** Dengue Serotype identified over Years

	2003	2004	2005
Total Cases(n) duration of fever <5 days	42	4	39
Culture Positive	8	2	5
RT PCR Positive	8(only on culture +ve)	2	17
Dengue Serotype By IFA & RT-PCR	2 dengue-1 2 dengue-2 3 dengue-3 1 dengue-4	2 dengue-1	1 dengue-1 14 dengue-3 2 dengue-1 plus dengue-3

## Discussion

Dengue is emerging as a major public health problem in India. Since the first epidemic in Kolkata during 1963–64 many places in India have been experiencing dengue infection [6]. One of the largest outbreaks in North India occurred in Delhi and adjoining areas in the year 1996. The 1996 epidemic was mainly due to dengue-2 virus [2,3]. Following this in the post epidemic period, 1997, dengue-1 virus activity was seen in Delhi [7]. Thereafter, in the year 2003 another outbreak occurred in Delhi and all four dengue virus serotypes were found to be co-circulating [4,5]. However, dengue-3 was reported to predominate in certain parts of North India in 2003[8].

In the following year (2004 and 2005) though no outbreak occurred in Delhi, definitely higher number of cases than usual were referred to our laboratory for testing. The seasonality of transmission of dengue with increased activity in the post monsoon season was seen in the present study; in accordance with the reported patterns of dengue transmission [9]. Even in the post-epidemic period (2004& 2005) increased dengue virus activity was seen in post monsoon period September to November with peak in the second and third week of October. Similar observation was seen in the year 1997 following 1996 epidemic [7]. These findings indicate that during epidemic and non-epidemic years dengue infections are mostly seen in post monsoon season hence preventive measures should be in full swing at the very onset of the monsoon.

Age wise distribution of the seropositive cases in all 3 years shows that statistically significant number of cases were in older age group (>10 yrs) as compared to the younger age group (≤ 10 yrs) (p value = <0.001). This observation is quite in accordance with our previous reported study [4] and with other studies from Delhi [10]. However many studies from South India [11] found children more susceptible to infection than the adults.

The predominant dengue virus serotype seen in the year 1997, following dengue-2 epidemic in 1996 was dengue-1[7]. In this study in the year 2004, dengue-1 was found to be circulating following 2003 outbreak, which involved all four serotypes. In the year 2005, however, in majority of cases dengue-3 was identified. dengue-2 and dengue-4 were not identified in 2005 indicating that dengue-3 seems to have replaced dengue-2 and 4 to establish itself as the predominant strain in Delhi.

Over the past two decades, dengue-3 has caused unexpected epidemics of DHF in Srilanka, East Africa and Latin America [12]. Emergence of dengue-3 has also been reported in 2003 as well as 2004 from certain parts in North India [8,13,14].

The present study reports the emergence of dengue-3 as the predominant serotype in Delhi in the year 2005. Further studies regarding the molecular characterization of these dengue-3 viruses are underway. Epidemiology of dengue infection in Delhi is rapidly changing face, with frequency of outbreaks increasing, even as dengue establishes itself as endemic disease. The need of the hour is to characterize the circulating serotypes of dengue virus in our community and understand the evolutionary processes influencing the dengue virus, as this is expected to impact on vaccination strategies for future.

## Conclusion

The year 2003 witnessed an outbreak after 1996 in Delhi. All four dengue serotypes were seen circulating but in 2005 complete pre dominance of dengue-3 was seen. The demographic picture of serologically confirmed cases in all three years remained almost the same with predominant age group involved as 21–30 years and maximum cases being seen in the post monsoon season of October.

## Methods

### Specimens

A total number of 1820 (Table 1) acute phase clotted blood samples collected from clinically suspected cases of dengue virus infection, coming to the various outpatient departments, emergency services and admitted patients at AIIMS, were tested for dengue specific IgM antibodies when the duration of fever was ≥5 days. When the duration of fever <5 days, acute phase clotted blood samples (n = 85) were collected on ice and transported to the virology laboratory in cold condition for viral isolation and reverse transcriptase polymerase chain reaction (RT-PCR). The Ethics committee of the institution approved this study.

### Virus isolation

Virus isolation was carried out in the C6/36 clone of *Aedes albopictus *cell lines as described by Broor et al (1997)[2]. Briefly, one in ten dilution of each serum sample (duration of fever <5 days) was inoculated in duplicate on a confluent monolayer of C6/36 cell line and were incubated at 25°C for 10 days. On the 10^th ^day, one tube was frozen at -70°C and cells from the other tube were harvested and cell spots were made on Teflon coated slides from each sample. Uninfected clone of *Aedes albopictus *cell line was used as negative control and cell lines infected with dengue virus 1 to 4 (Obtained from the National Institute of Virology, Pune, India) were included as positive controls in each run. IFA was performed on these spots using monoclonal antibodies to dengue 1–4 (provided by Dr. D.J. Gubler, then at CDC, Atlanta, during the 1996 outbreak). If IFA was negative for dengue viruses on first passage, a blinded second passage was made and cells were again harvested on 10^th ^day for IFA. If the IFA was still negative for dengue viruses, then the sample was declared negative for virus isolation [15].

### IgM antibody capture enzyme linked immunosorbent assay (MAC-ELISA)

Serum sample (duration of fever ≥5 days) were screened for the presence of IgM antibodies using IgM capture ELISA PanBio, Australia) following the manufactures protocol. OD was measured at 450 nm using an ELISA reader (Labsystems Multiskan Plus Finland).

### Dengue specific reverse transcriptase polymerase chain reaction (RT-PCR)

Dengue viral RNA was isolated from the serum samples using the QIA amp viral RNA mini kit (Qiagen, Germany) as per manufactures protocol. The RT-PCR assay employed in this study could distinguish the 4 dengue serotypes by the size of the products as described by Lanciotti et.al [16]. This includes a step of RT-PCR using a highly conserved primer pair, D1 (forward) and D2 (reverse) and a step of second-round PCR using the primer D1 and 4 serotype-specific primers, TS1, TS2, TS3 and TS4.

The expected size of the RT-PCR products is 511 bp (D1 and D2) (external PCR product) and 482-bp (D1 and TS1 for dengue-1), 119-bp (D1 and TS2 for dengue-2), 290 bp (D1 and TS3 for dengue-3) and 392-bp (D1 and TS4 for dengue-4). The products were electrophoresed through 2% agarose gel, stained with ethidium bromide and examined under ultraviolet light using a digital gel documentation system.
